# Helix tack suspension for esophageal stent fixation

**DOI:** 10.1016/j.vgie.2022.02.014

**Published:** 2022-05-13

**Authors:** Roberto P. Trasolini, James K. Stone, Neal A. Mehta, Mandeep S. Sawhney, Tyler M. Berzin

**Affiliations:** Center for Advanced Endoscopy, Beth Israel Deaconess Medical Center and Harvard Medical School, Boston, Massachusetts

**Keywords:** OTS, over the scope, TTS, through the scope

## Abstract

Video 1 Case description, description of suture pattern and technique, and video demonstrating technique in vivo, follow-up endoscopy, and conclusions.

Case description, description of suture pattern and technique, and video demonstrating technique in vivo, follow-up endoscopy, and conclusions.

## Introduction

Fully covered self-expanding metal stents were developed as a removable prosthesis for esophageal obstruction. They are highly effective at relieving dysphagia and are covered with an impermeable membrane to prevent tissue ingrowth. Unfortunately, these stents carry a significant risk of migration,^1^ with risk for subsequent adverse events including intestinal obstruction^2^ and, rarely, perforation.^3^ If the esophageal stricture is endoscopically nonpassable, there is also a risk of requiring surgical removal.^4^ The overall migration rate for fully covered self-expanding metal stents in benign esophageal strictures is 30%, and clinically significant migration occurs in 17%.^1^

In part due to migration risk, esophageal stents are not recommended as first-line therapy in benign esophageal strictures but can be used as an adjunct^5^; in some institutions, they are kept in place for 3 to 6 months based on clinical experience and safety record.^6^ Multiple strategies for stent fixation have been tried to reduce the migration rate, including through-the-scope (TTS) hemostatic clips,^7^ external fixation to a patient’s nares or ear,^8^ over-the-scope (OTS) clips,^9^ and endoscopic sutures using an OTS device.^10^ There remains a need for a fixation method that is effective while optimizing patient comfort, ease of deployment, and acceptable cost. Because of limitations in current options for stent fixation, we elected to establish the feasibility of a novel method of stent fixation in discussion with the patient using TTS suturing. TTS suturing relies on capture of tissue by helical screws, with tissue approximation via tension placed on an attached suture. The technique for general application of TTS sutures is well described in a recent video article.^11^

## Case report

**Figure 1:**
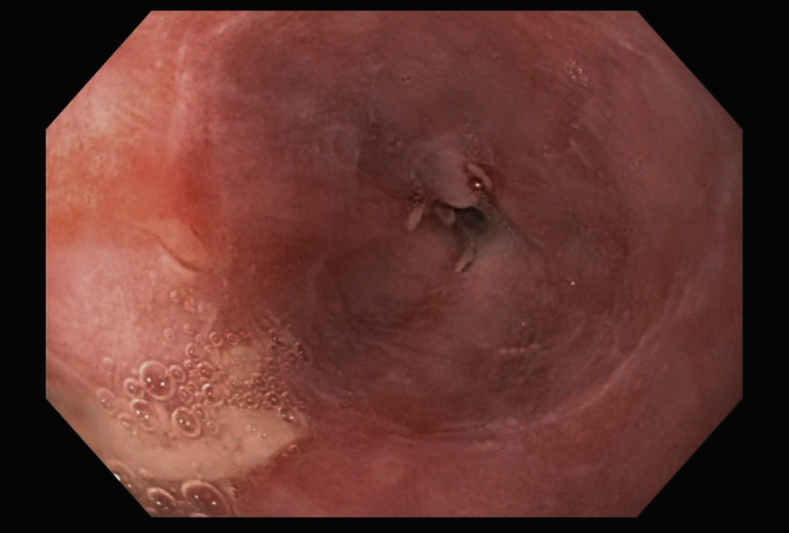
Esophageal stricture before stenting.

A 44-year-old man with a history of treated esophageal lymphoma presented with severe dysphagia. After previous treatment with chemotherapy and radiation, a narrow, benign, 3-cm stricture had developed in the distal esophagus and was refractory to standard endoscopic therapies (Fig. 1). The decision was made to place a fully covered metal stent, given failure of previous therapies, but we had concerns related to migration. Based on positive experiences with TTS suturing, we decided to fix the stent using a novel technique, which we have termed helix tack suspension, using a TTS helical suturing device. For this procedure, we used TTS helix tacks with attached polypropylene suture to “suspend” the stent by its proximal retrieval suture (Video 1, available online at www.giejournal.org). This technique was chosen to facilitate ease of removal, given uncertainty about stent tolerance and a lack of established protocol for removing helix tacks. Early reports suggest helix tacks remain in place for weeks to months.^12^

**Figure 2:**
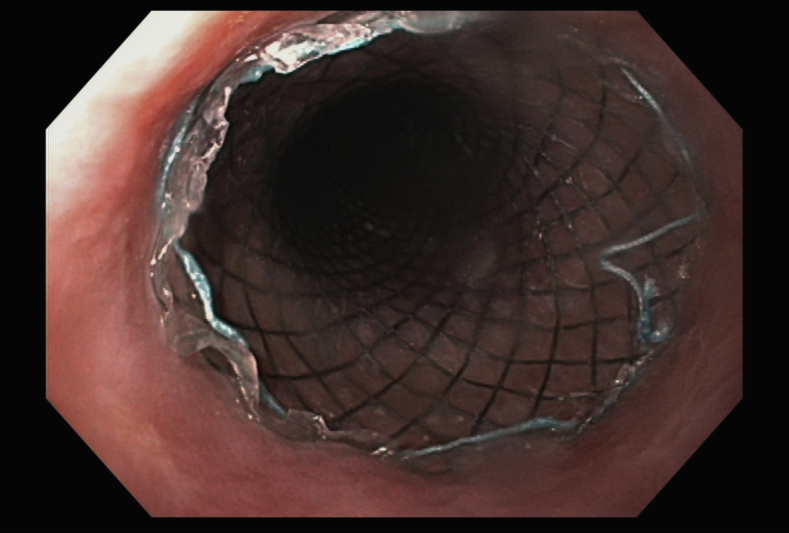
Esophageal stent placement.

**Figure 3:**
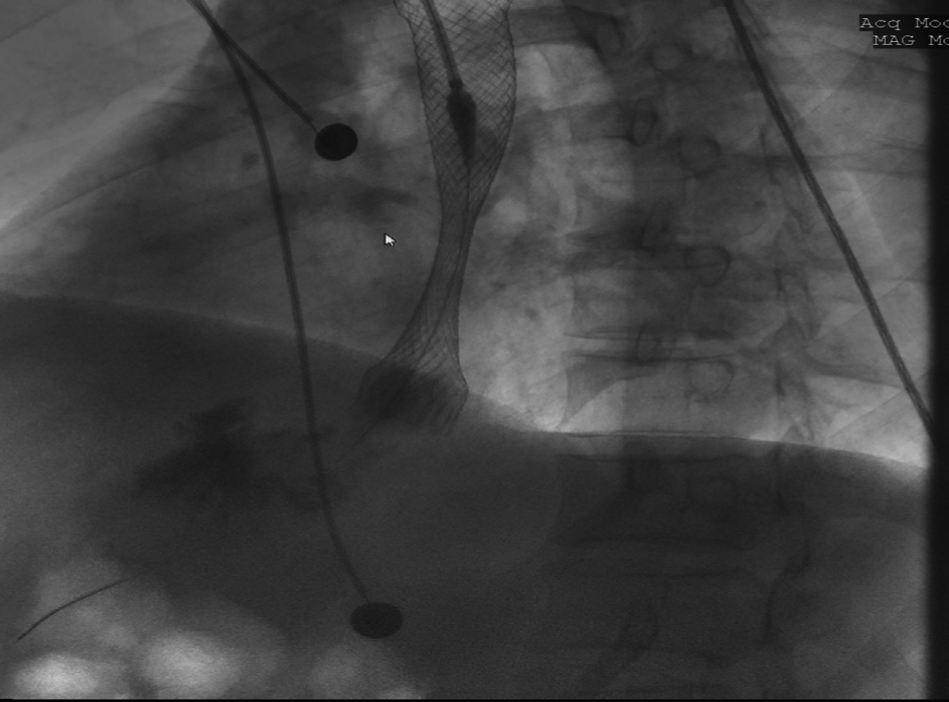
Fluoroscopic image of stent deployment.

**Figure 4:**
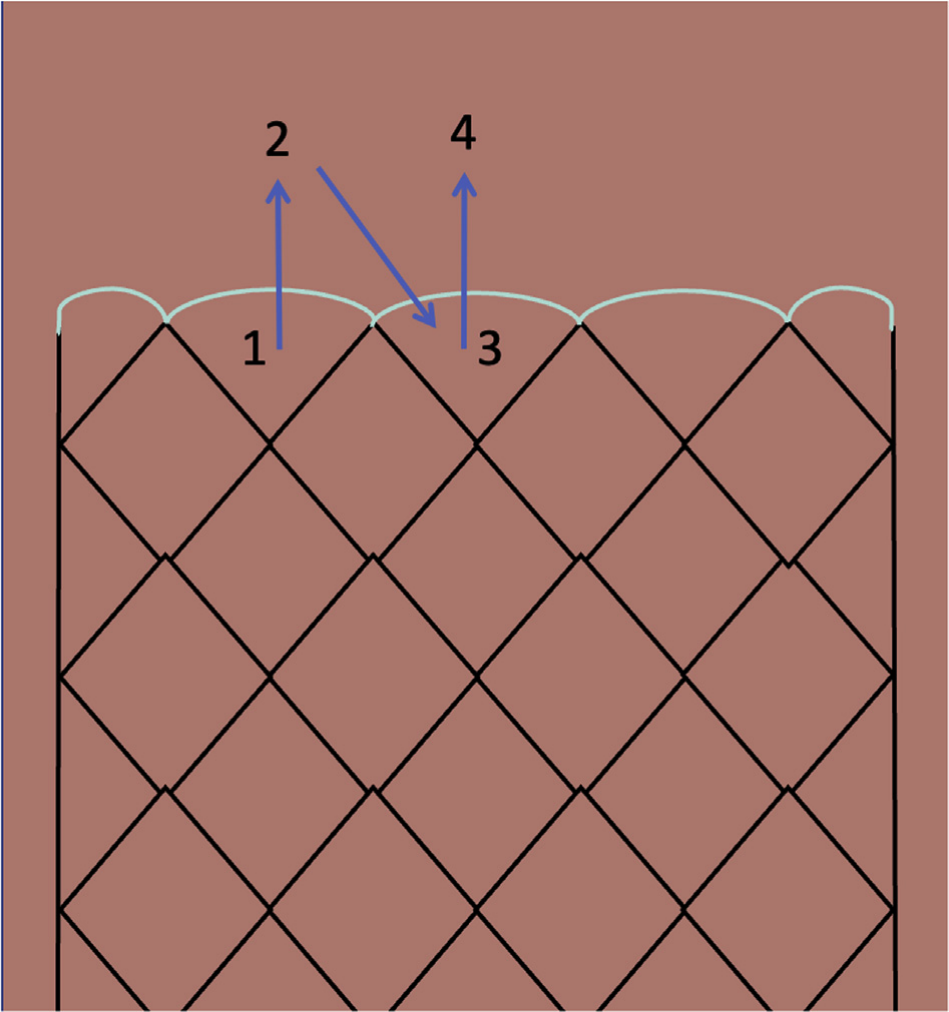
Graphical depiction of suture pattern. Green line represents stent retrieval suture.

**Figure 5:**
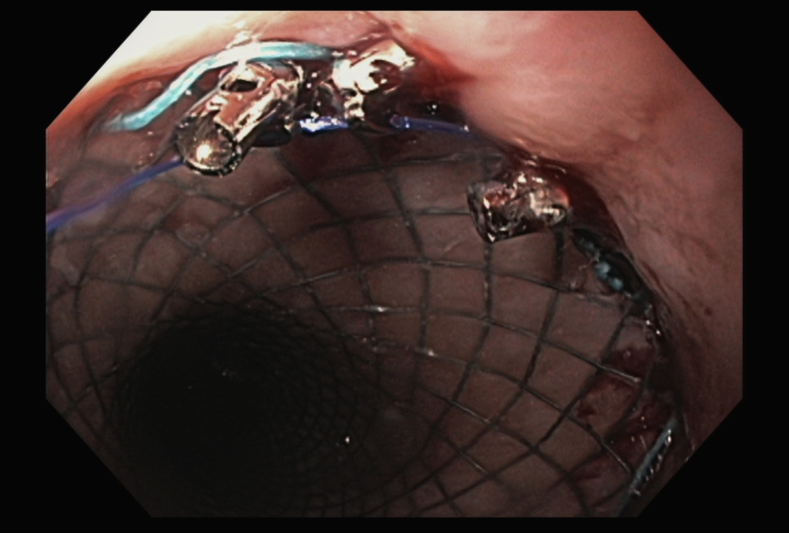
Helix tack suture deployment.

**Figure 6:**
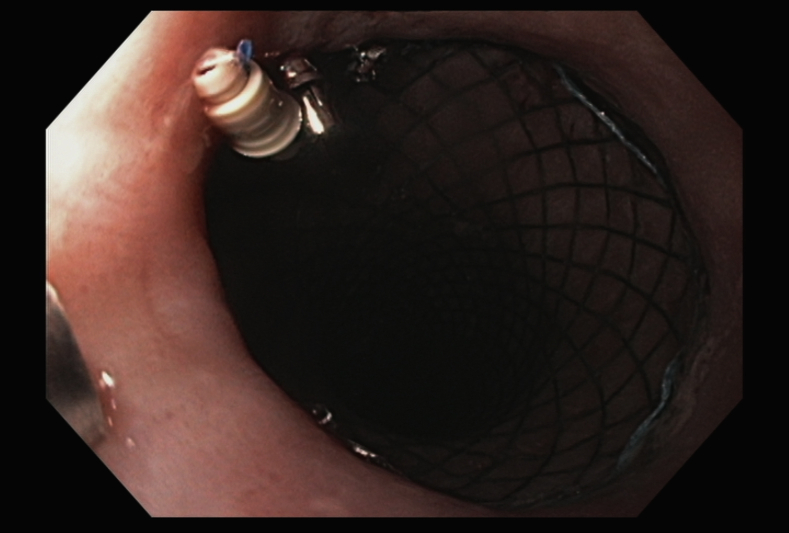
Final suture appearance. Note minimal tension on suture.

**Figure 7:**
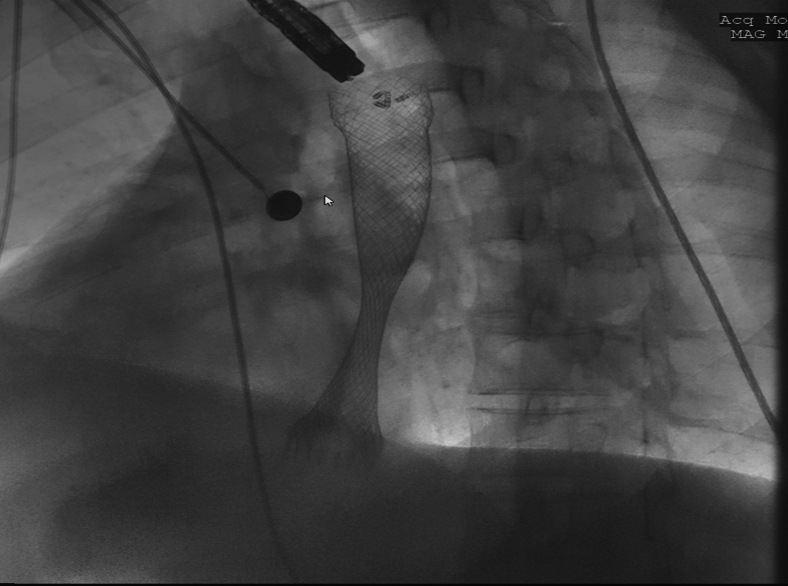
Fluoroscopic image of helix tack stent fixation.

We began by deploying a fully covered esophageal stent (WallFlex; Boston Scientific, Marlboroug, Mass) under fluoroscopy with good results both endoscopically (Fig. 2) and fluoroscopically (Fig. 3). A helix tack suturing device (X-tack; Apollo Endosurgery, Austin, Tex) was then deployed TTS using an “N” type suture pattern to capture the retrieval suture at the proximal end of the stent (Figure 4, Figure 5). We elected to use this placement with minimal tension to maximize tissue capture and prevent deformation of the stent as well as to facilitate suture removal if needed. The final result was satisfactory both endoscopically (Fig. 6) and fluoroscopically (Fig. 7).

**Figure 8:**
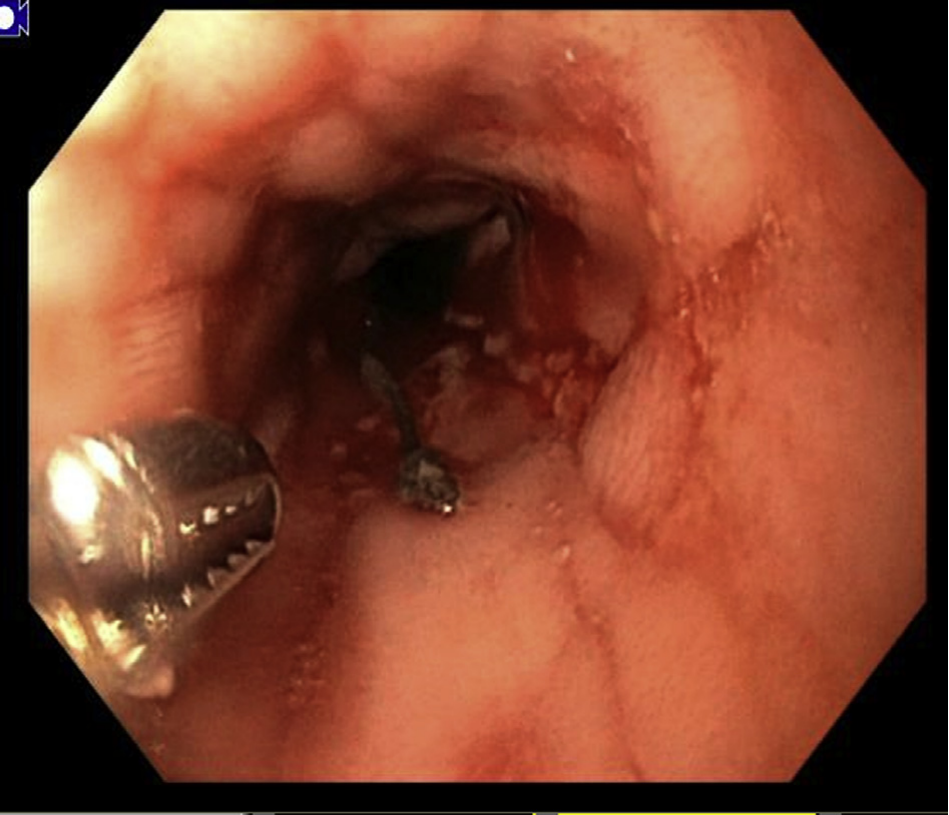
Proximal esophagitis and narrowing at 3-month follow-up endoscopy.

At 1 month follow-up, the patient was tolerating an enteral stent diet with no discomfort. After 3 months, the patient contacted us with progressive dysphagia and inability to tolerate more than thin liquids. Endoscopy showed severe ulceration and narrowing above the stent, with the previously placed helix tacks no longer visible and the retrieval suture degraded (Fig. 8). No tacks were seen on fluoroscopy. The stent was removed uneventfully with rat-tooth forceps, and acid suppression was optimized. After 1 week without a stent, the original stricture recurred to its previous diameter. A new stent was placed and fixed with full-thickness sutures using the Apollo Overstitch device, with plans to reassess in 3 months given tolerance of the previous stent.

## Discussion

Multiple techniques have been described for esophageal stent fixation; however, current techniques are limited in efficacy, patient comfort, or ease of deployment. Costs are also a consideration, with the cost of OTS suturing devices being twice that of TTS helix tack suturing.^12^ Endoscopic TTS suturing has been recently described with placement through the stent covering.^12^ Our technique differs in that the removal suture is targeted, which is a potentially useful strategy when ease of removal is a priority. Use of a TTS suturing device for stent fixation may provide improved efficacy compared to TTS clips, improved patient comfort compared to external fixation and decreased costs, and easier removability compared to OTS clips. OTS suturing devices have good durability and effectiveness but are expensive, and expertise with their use is currently limited. In our experience, TTS suturing has a short learning curve and good tissue capture, with promising potential for applications where full-thickness bites are not required or desirable.

## Disclosure

*Dr Berzin is a consultant for Boston Scientific, Medtronic, and Conmed. All other authors disclosed no financial relationships.*

## Supplementary data

Video 1 Case description, description of suture pattern and technique, and video demonstrating technique in vivo, follow-up endoscopy, and conclusions.
